# Bleaching as a complement to fluoride-enhanced remineralization or resin infiltration in masking white spot lesions^*^

**DOI:** 10.1590/1678-7757-2024-0097

**Published:** 2024-09-20

**Authors:** Talita Portela PEREIRA, Karin LANDMAYER, Bruna de Oliveira IATAROLA, Mariele VERTUAN, Ana Carolina MAGALHÃES, Luciana Fávaro FRANCISCONI-DOS-RIOS

**Affiliations:** 1 Universidade de São Paulo Faculdade de Odontologia Departamento de Dentística São Paulo Brasil Universidade de São Paulo, Faculdade de Odontologia, Departamento de Dentística, São Paulo, Brasil.; 2 Universidade de São Paulo Faculdade de Odontologia de Bauru Departamento de Ciências Biológicas Bauru Brasil Universidade de São Paulo, Faculdade de Odontologia de Bauru, Departamento de Ciências Biológicas, Bauru, Brasil.

**Keywords:** Color, Fluoride, Resin infiltration, Tooth bleaching, Dental enamel, Dental caries

## Abstract

There are many suitable strategies for addressing caries, which is an ongoing worldwide problem. Although white spot lesions (WSLs) can be either remineralized naturally or treated with non- or micro-invasive strategies, their whitish and opaque appearance may persist.

**Objective:**

To evaluate the effects of tooth bleaching as a complement to fluoride-enhanced remineralization or resin infiltration in masking WSLs, as well as in enamel surface roughness relative to that of the adjacent enamel.

**Methodology:**

Flattened rectangular bovine enamel fragments (6×3×~2.9 mm length, width and thickness) were divided into six groups (L/N, F/N, F.BL/BL, I/N, I.BL/BL, N/N; n=15). Treatments applied to the 3×3 mm left half included: L (Lesion) - WSL simulation with 50 mM acetate buffer, 96 hours, 37ºC; F (Fluoride) - WSL treatment with 2% NaF neutral gel, 1x/week, 8 weeks; I (Infiltration) - WSL treatment with H_3_PO_4_ 37%/10 s; Icon^®^-Dry/30 s; Icon^®^-Infiltrant/3 min+1 min; N (Nothing) - sound enamel/control. Treatments applied to both halves after F and I included: BL (Bleaching) - Opalescence Boost 40%, 3×/20 min each; N (Nothing) - control. The differences in color (ΔE_00_, ΔL, Δa, Δb) and surface roughness (ΔRa) between the left and right halves were measured. Kruskal-Wallis/post-hoc tests were applied to ΔE_00_, ΔL, Δa and ΔRa, and 1-way ANOVA/Tukey tests to Δb (α=0.05).

**Results:**

The factor under study significantly influenced ΔE_00_ (p=0.0001), ΔL (p=0.0024), Δb (p=0.0015), and ΔRa (p<0.001), but not Δa (p=0.1592). Both fluoride-enhanced remineralization and resin infiltration were able to mask WSL, regardless of subsequent bleaching. However, when bleaching was performed, ΔE_00_ median values did not exceed the acceptability threshold for color difference. Only resin infiltration reduced ΔRa between WSL and the adjacent enamel.

**Conclusions:**

Both remineralization and infiltration, particularly if complemented by bleaching, fostered satisfactory esthetic results. Only infiltration without bleaching led to really good results in surface roughness.

## Introduction

A recent investigation into the burden of untreated dental caries in 204 countries and territories over 30 years has revealed a significant increase in its incidence, prevalence, and number of years lived with disability, especially among groups with high free sugar intake and those in lower socioeconomic positions.^1^ Many strategies are suitable for addressing the caries process as well as its signs and symptoms. While invasive/restorative interventions are generally indicated for active cavitated lesions, no treatment might be the clinical practice for inactive non-cavitated and cavitated lesions (except for reasons of form, function or esthetics). Furthermore, non- or micro-invasive strategies might help with active non-cavitated carious lesions, or white spot lesions (WSLs).^2^

Active WSLs are typically subsurface, presenting a pseudo-intact surface layer over the body of the lesion with plenty of pores. These may be filled with saliva/water, which has a refractive index of ~1.33, or air, which has a refractive index of ~1.0. Both differ from that of the hydroxyapatite, which is ~1.65. The greater the difference between the refractive indices, the whiter the appearance of the lesion^3^, which may lead to esthetic discomfort when located in anterior teeth.^4^

Treatment of WSLs should ideally arrest them and simultaneously favor esthetics, thus avoiding cavitation and lessening opacity and the possibility of discoloration.^5^ Although fluoride-enhanced remineralization is an effective non-invasive treatment for arresting WSLs,^6^ its esthetic result may not be successful. In general, WSLs remain visible after this procedure due to the rapid precipitation of ions in their outer portions. As a result, the ions cannot penetrate into the inner portions of the lesion body and the subsurface remains porous.^7^

On the other hand, resin infiltration in WSLs has been proven to stop mineral loss and reduce their whitish appearance. This micro-invasive strategy consists of infiltrating a low-viscosity TEGDMA-based material with a high penetration coefficient into the intercrystalline spaces of demineralized enamel after etching the pseudo-intact surface layer.^8^ The material replaces saliva/water into the lesion body pores, granting the WSL mechanical support and an optical appearance similar to that of the adjacent sound enamel, as its refractive index is ~1.52.^9^

Nonetheless, WSLs masking can be challenging, and its whitish aspect tends to be only partially masked due to the histopathologic lesion features and the high sensitivity of the technique.^10^ Some amount of remaining opacity after enamel resin infiltration seems to be inevitable, possibly due to incomplete replacement of the air that fills the total volume of enamel pores by the material.^11^ Application of the infiltrant itself over the WSL was suggested to cause a low outward air flow rate, which is part of the typical flow competition that occurs during the infiltration of liquids into dry porous hard materials.^11^ Therefore, doubts remain as to how to achieve better esthetic results without furthering loss of tooth structure and impairing enamel surface roughness, when already remineralized or infiltrated WSLs do not completely disappear.

Bleaching, for instance, is a non-invasive strategy to treat discolored teeth which has been highly recommended as the first attempt to reduce the contrast between white spots and the adjacent sound tooth structure.^12^ Bleaching plus resin infiltration is widely accepted to mask fluorosis and other enamel defects.^13,14^ However, Jacob, et al.^15^ (2023), on their study of bleaching on color and surface topography of teeth with enamel caries differently treated, were categorical in establishing as their background that bleaching is not recommended on teeth with demineralized carious lesions. There were also concerns from a respected consultant and employees of the company that holds the patent for the only resin infiltrant commercially available on the recommendation of bleaching before infiltration, as in clinical practice, this sequence is not always reasonable.^16^ Recognizing that the interaction between the bleaching agents and the resin-infiltrated lesions themselves is an area of interest, they just evaluated the effect of bleaching after resin infiltration regarding surface roughness and color using bovine incisors.^16^ However, bleaching as a complement to resin infiltration or to fluoride-enhanced remineralization in masking WSLs (which is different from understanding the WSL itself before and after a given treatment or combination of treatments) was not extensively evaluated.

In this context, clinicians should have evidence-based alternatives to address esthetic concerns remaining from the partially successful previous treatments of WSLs.^17^ This, as well as the need to justify further randomized clinical trials on masking WSLs, were the reasons for the proposal of this study.

Therefore, the objective was to evaluate the ability of bleaching after fluoride-enhanced remineralization and resin infiltration, as well as that of each of them not followed by bleaching, to mask WSLs in bovine enamel and to influence its surface roughness, relative to that of the adjacent sound enamel. The null hypothesis tested was: neither bleaching after fluoride-enhanced remineralization and resin infiltration, nor each of them not followed by bleaching, would affect the masking of WSLs or its roughness relative to adjacent sound enamel.

## Methodology

### Sample size calculation

Considering that an ΔE_00_=6.28±0.531 was previously verified between WSLs and adjacent enamel,^18^ and 0.8 is the CIEDE2000 color difference perceptibility threshold,^19^ sample size was calculated (http://estatistica.bauru.usp.br/calculoamostral/) using an estimated standard deviation of 0.531 and an effect size of 0.8, plus alpha and beta errors of 5 and 20%. It was found that n=14, but n=15 was selected for each group.

### Specimens’ preparation and distribution in the experimental groups

After the Ethics Committee on Animal Use exempted this research project from being analyzed (CEUA/FOUSP #025/2019), 150 bovine incisors were obtained. Prior to the specimens’ preparation, cracked or stained teeth were excluded from the study and then each of the pertinent ones had the crown sectioned in a 6×3×~2.9 mm length, width and thickness rectangular fragment using a precision cutting machine (Isomet Low Speed Saw; Buehler Ltd., Lake Buff, IL, USA). The dentin and enamel of the fragments were flattened, and the enamel also polished, in a metallographic polisher (EcoMet; Buehler Ltd., Lake Buff, IL, USA) to a thickness of approximately 1.6 mm and 1.3 mm, respectively. The specimens were immersed in distilled water for 10 minutes in an ultrasonic bath (Shenzhen Codyson Electrical Co., Ltd., CHN, Guangdong, China) and those still cracked were excluded from the study. Then, specimens were numbered and submitted to surface microhardness analysis (Knoop Hardness Number [KHN]) using a microhardness tester (HMV-G21DT, Shimadzu Co. Tokyo, Japan) with a Knoop indenter (50 g/10 s).^20^ Five indentations were made to determine the mean and standard deviation of the microhardness value. Blocks with a standard deviation greater than 10% of their individual mean microhardness and individual mean microhardness greater or less than 10% of the mean microhardness calculated for all blocks (324.6±13.3) were excluded.

A total of 90 specimens were selected for distribution by stratified randomization (Excel 16.0; Microsoft Corporation, Redmond, WA, USA) into six experimental groups (n=15):

L/N: Lesion without treatment (left half)/Nothing (right half);

F/N: Fluoride treatment (left half)/Nothing (right half);

F.BL/BL: Fluoride treatment + bleaching (left half)/Bleaching (right half);

I/N: Infiltration treatment (left half)/Nothing (right half);

I.BL/BL: Infiltration treatment + bleaching (left half)/Bleaching (right half);

N/N: Nothing/Nothing (control group).

**Figure 1:**
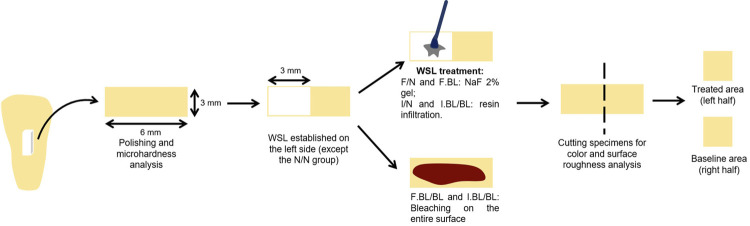
Work flowchart

The work flowchart (Figure 1) summarizes the conducted procedures.

Remaining specimens were used for the validation of the protocol for simulating the WSLs or for pilot tests, or were stored in distilled water so that they could replace any specimen previously selected in case of technical artifacts which could lead to exclusion from the study.

### Validation of protocol for simulating white spot lesions

In nine specimens with surface microhardness consistent with those of the 90 specimens in the experimental groups, a central window measuring 3×3 mm was determined in order to achieve contact with the demineralizing solution for 32, 64 or 96 hours (n=3) (50 mM acetate buffer; 1,28 mmol/L of Ca(NO_3_)_2_.4H_2_O, 0,74 mM NaH_2_PO_4_.2H_2_O, and 0,03 ppm F, pH 5.0, 37ºC).^21,22^

**Figure 2:**
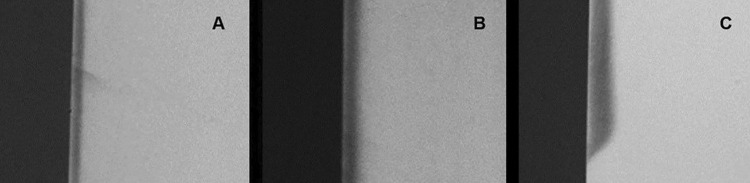
Representative TMR images of most slices of demineralized specimens for 32 h (A), 64 h (B) and 96 h (C)

All the specimens were transversally sectioned and polished to obtain slices of 80-100 µm each. These slices were then affixed to specific plates and exposed to radiation using a Transversal Microradiography system (TMR; TMR 1.25e, Inspector Research BV, Amsterdam, Netherlands).^23^ A transmitted light microscope with a 20× objective (Axioplan; Zeiss, Oberkochen, Germany) and a camera (XC-77CE, Sony, Tokyo, Japan) were used to observe whether the lesion was subsurface. The TMR 1.25e system software was used to calculate the integrated mineral loss (ΔZ, %vol.μm) by subtracting the percentage of mineral volume of sound enamel (87%) from that percentage of the demineralized enamel, multiplied by the lesion depth (μm). Lesion depth (LD, µm), the integrated mineral loss (∆Z, vol. µm), and the average mineral loss over the lesion depth (R, vol%) were obtained. Data from specimens immersed for 32, 64 and 96 hours are presented in Table 1, and their representative images in Figure 2.

**Table 1:** Integrated mineral loss (ΔZ, % vol.µm), lesion depth (LD, µm), and mean mineral loss (R, %vol) of the specimens immersed in the demineralizing solution, plus mean and standard deviation for each of the immersion durations.

Immersion duration	ΔZ (vol%.μm)	LD (μm)	R (%vol)
32h	2139.4±393.6	85.0±13.9	25.3±1.3
64h	3694.4±300.9	138.4±10.3	26.4±1.2
96h	5525.4±395.9	139.4±6.2	39.5±4.4

### Simulation of WSL and specimens’ treatment

The right half, the lateral and dentin surfaces of each specimen in the L/N, F/N, F.BL/BL, I/N, and I.BL/BL groups were coated with three layers of cosmetic nail varnish (Colorama Longa Duração Extra Brilho; L’Oréal Brasil Comercial de Cosméticos Ltda., Rio de Janeiro, RJ, Brazil). This ensured that only the left half had artificial WSL, according to the validated protocol. The specimens were immersed in the demineralizing solution for 96 h, since the greatest depth was verified for this amount of time. Once the artificial WSLs were established, the following treatments were conducted:

### L/N: No treatment on the left half surface (with the WSL).

F/N and F.BL/BL: 2% NaF neutral gel (SS White Artigos Dentários Ltda., Rio de Janeiro, RJ, Brazil) for 1 min on the left half surface (with the WSL). After each application of the gel, the specimens were rinsed with distilled water and stored in 6.3 mL of artificial saliva (22.1 mmol/L hydrogen carbonate, 16.1 mmol/L potassium, 14.5 mmol/L sodium, 2.6 mmol/L hydrogen phosphate, 0.8 mmol/L boric acid, 0.7 mmol/L calcium, 0.2 mmol/L thiocyanate and 0.2 mmol/L magnesium; pH between 7.4 and 7.8), which was replaced daily.^24,25^

I/N and I.BL/BL: 37% phosphoric acid (Condac 37, FGM Dental Group, Joinville, SC, Brazil) for 10 s^26^on the left half surface (with the WSL). After 30 s of air-water spray rinsing and drying, Icon^®^-Dry was applied for 30 s and air-dried. Finally, Icon^®^ infiltrant was applied twice: the first time for 3 min, and the second for 1 min. After excesses were removed, each application was light-cured for 40 s (Radii-cal, SDI, Bayswater, VIC, Australia). Polishing was performed with an abrasive rubber cup (Enhance Finishing System, Dentsply Caulk, Milford, DE, USA) for 20 s at low speed.

F.BL/BL and I.BL/BL: Bleaching with 40% hydrogen peroxide gel on the entire surface of the specimens (Opalescence Boost 40% Hydrogen Peroxide, Ultradent, South Jordan, UT, USA). A 0.5-1.0 mm thick layer of gel was applied, and three applications were made for 20 minutes each.

* In the N/N group, both halves were only abraded and polished (control group).

### Evaluation of color difference between WSL and adjacent enamel

**Figure 3:**
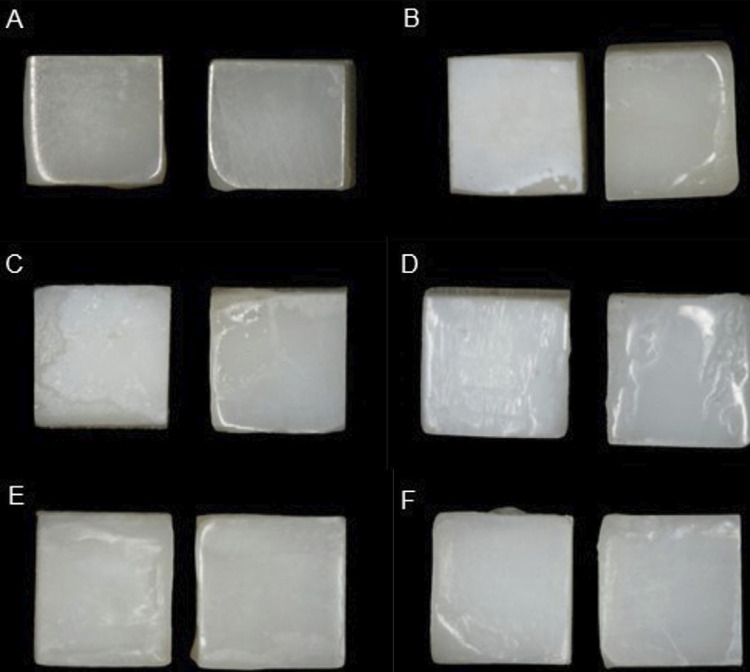
Visual aspect of a representative specimen from each of the groups A) N/N (control); B) L/N; C) F/N; D) F.BL/BL; E) I/N; F) I.BL/BL

Specimens were stored in distilled water (37ºC, 24 h) and then sectioned into two 3×3 mm halves (Isomet Low Speed Saw; Buehler Ltd., Lake Buff, IL, USA). After gently drying with an absorbent paper, each half-specimen was placed into a polytetrafluoroethylene holder with a 3×3 mm reading window, and color reading was conducted using a colorimetric reflectance spectrophotometer (CM 3700A, Konica Minolta, Osaka, Japan). Color and spectral distribution were measured according to the coordinates L*, a* and b* established by the Commission International de l’Eclariage (CIE). Following settings were used: 360-740 nm wavelength light, standard illuminant D65, 2º observer angle and a white background. ΔE_00_ (using CIEDE2000 color difference formula; $\Delta E_{00}=\{{\left[\Delta L/\left(K_{L}S_{L}\right)\right]}^{2}+[\Delta C/{{K_{C}S_{C}]}^{2}+{\left[\Delta h/\left(K_{h}S_{h}\right)\right]}^{2}+\Delta R\}}^{1/2}$ , ΔL, Δa, and Δb were determined by subtracting the adjacent enamel data (right half) from the WSL data (left half). Figure 3 shows the visual aspect of a representative specimen from each of the experimental groups.

### Evaluation of surface roughness difference between WSL and adjacent enamel

Sites corresponding to the center of the surface of each half-specimen and 1.5 mm up and down were scanned with an optical profilometer (Proscan 2100 – Sensor Model S11/03, Scantron, Venture Way, Tauton, UK). The following settings were used: cut off = 0.8 mm, surface filter = 99, step size X = 0.002 / number of steps X = 2000, and step size Y = 0.001 / number of steps Y = 0. With the help of the system software (Proscan Application software v. 2.0.17, Scantron, Venture Way, Tauton, UK), arithmetic average roughness (Ra) was determined and the mean of the three readings assigned as the Ra for each half-specimen. ΔRa were determined by subtracting the adjacent enamel data (right half) from the WSL data (left half).

### Statistical analysis

The Shapiro-Wilk and Levene tests were applied to evaluate distribution of data. The data for ΔE_00_, ΔL and Δa did not respect assumptions of normality, and the ΔE_00_ and ΔRa of homogeneity. Thus, the Kruskal-Wallis and Dunn tests were applied. The data for Δb complied with both assumptions, and ١-way ANOVA and Tukey test were applied. Significance level was always 0.05, and the statistical program used was Statistica 13.5.17 (TIBCO Software Inc., Palo Alto, CA, USA).

## Results

**Figure 4:**
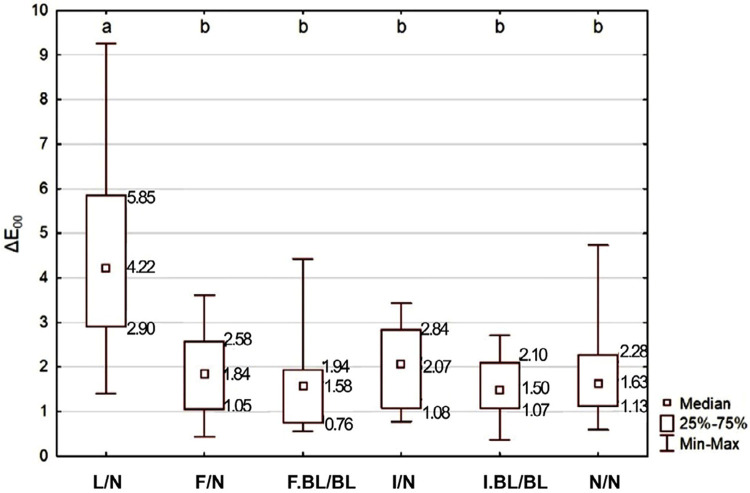
ΔE00 (total color change) depending on the WSL treatment, as well as the adjacent enamel.

The factor under study significantly influenced ΔE_00_ results (p=0.0001): the lesion without treatment (L/N) group differed from all other groups, which in turn did not differ from each other. WSLs thus contrasted with adjacent enamel, but both fluoride-enhanced remineralization and resin infiltration, followed or not by bleaching, masked them similarly. Median ΔE_00_ values when fluoride-enhanced remineralization and resin infiltration were not followed by bleaching, however, exceeded color difference perceptibility and acceptability thresholds (0.8 and 1.8, respectively)^19^ (Figure 4).

*Different letters indicate statistically significant difference between experimental groups

**Figure 5:**
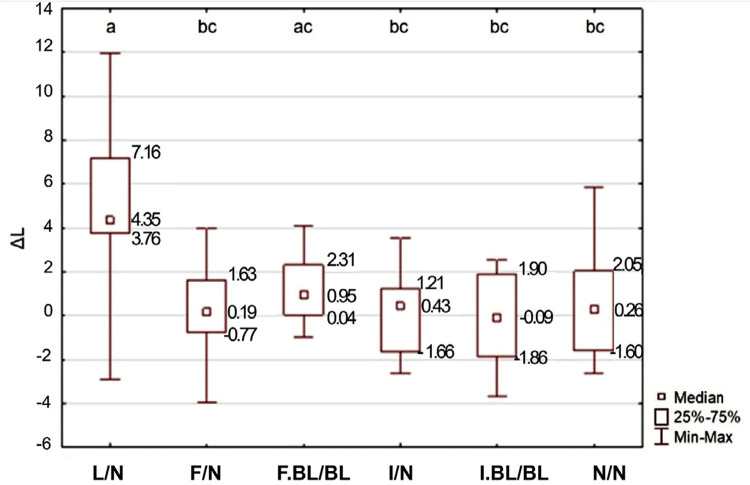
ΔL (*L coordinate – luminosity) depending on the WSL treatment, as well as the adjacent enamel.

Regarding ΔL, the factor under study also significantly influenced the results (p=0.0024): the lesion without treatment (L/N) group differed from the others, but the group with fluoride treatment plus bleaching (F.BL/BL) did not (p=0.79). All groups, except for the lesion without treatment (L/N) group, were not different from each other. The lesion without treatment (L/N) group showed the greatest ΔL, while the fluoride treatment plus bleaching (F.BL/BL) group showed intermediate values. The other groups showed the lowest values, which were equivalent to those of the control (N/N) group (Figure 5).

*Different letters indicate statistically significant difference between experimental groups

**Figure 6:**
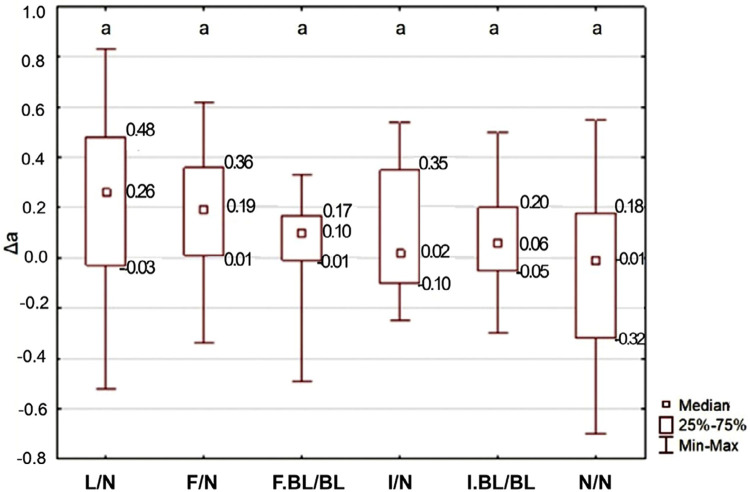
Δa (red[+]-green[-] coordinate) depending on the WSL treatment, as well as the adjacent enamel.

As for Δa, the factor under study did not significantly influence the results (p=0.1592): In the red[+]-green[-] coordinate, WSLs did not stand out from the adjacent enamel, regardless of its treatment and the subsequent bleaching (Figure 6).

**Figure 7:**
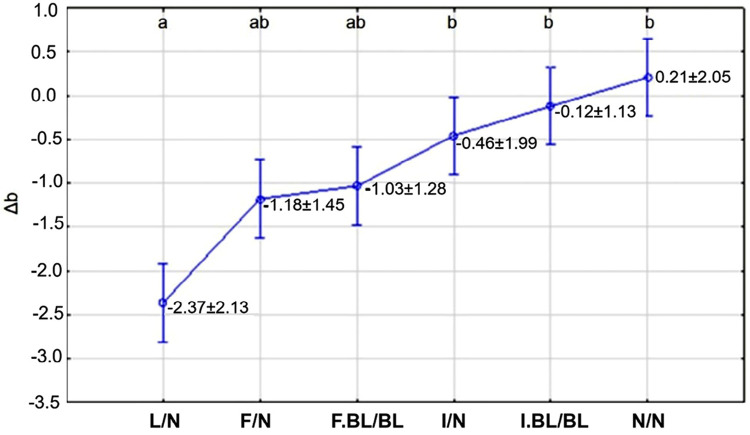
Δb (yellow[+]-blue[-] coordinate) depending on the WSL treatment, as well as the adjacent enamel.

About Δb, the factor under study significantly influenced the results (p=0.0015): the lesion without treatment (L/N) group was different from the other groups, except for the fluoride treatment (F/N) and fluoride treatment plus bleaching (F.BL/BL). All groups, other than the lesion without treatment (L/N) group, were not different among themselves. In the yellow[+]-blue[-] coordinate, WSL stood out from the adjacent enamel when it was not treated, but this did not occur when it was infiltrated, or infiltrated and bleached. An intermediate situation was found when the WSL was treated by fluoride-enhanced remineralization, complemented or not by bleaching (Figure 7).

*Different letters indicate statistically significant difference between experimental groups

**Figure 8:**
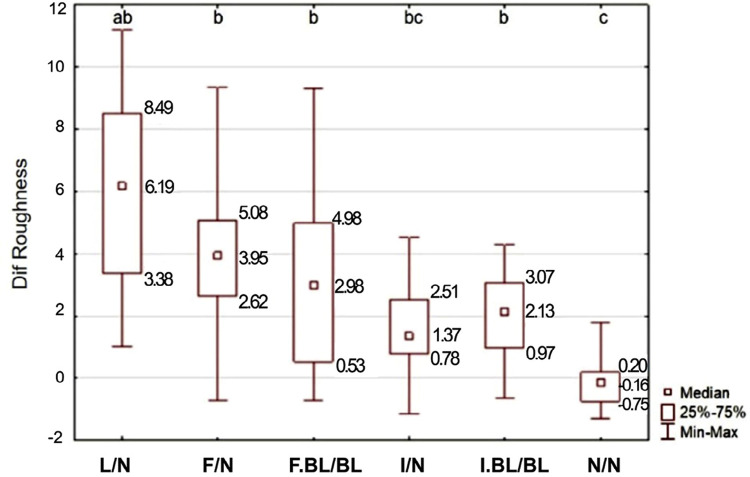
Difference in roughness (ΔRa) depending on the WSL treatment, as well as the adjacent enamel.

Finally, the factor under study significantly influenced ΔRa results (p<0.001): control (N/N) group was different from the other groups, except for infiltration treatment (I/N) group. Infiltration treatment (I/N) group was different from lesion without treatment (L/N) group. All groups, except for control (N/N) group, were not different among themselves. Only infiltration without bleaching reduced ΔRa between the WSL and the adjacent enamel as to it to be similar than that for the control (N/N) group (Figure 8).

*Different letters indicate statistically significant difference between experimental groups

## Discussion

In view of the presented results, the proposed null hypothesis was rejected, since bleaching after fluoride-enhanced remineralization and resin infiltration, and each of them not followed by bleaching, were able to mask the WSLs. Furthermore, resin infiltration not followed by bleaching was effective in minimizing the roughness difference between the WSL and the adjacent enamel.

In general, patients with WSLs feel dissatisfied with their teeth color due to their white appearance.^4^ In this study, this could be evidenced by the ΔE_00_ analysis, in which the enamel surface that contained the WSL highly contrasted with its adjacent sound surface. Specifically, there was a notable increase in luminosity (*L coordinates) and a blue tendency in WSL (*b coordinates - yellow[+]/blue[-]), which is consistent with previous studies.^25,27,28^ This fact can be justified by the loss of enamel mineral content, which reduces its translucency and increases its opacity.^27,29^

Treatments for WSLs ideally should arrest them, and whenever possible, improve their whitish appearance, so that the lesion becomes imperceptible relative to the adjacent sound enamel. However, most *in vitro* studies evaluate color differences of a same area, before and after the interventions,^25,27,30^ leading to a necessity for studies that compare the WSL color with that of the adjacent sound surface. This methodology was called in the literature “split-tooth”^28^, as the color analysis is conducted between two halves of a same specimen. Considering that this is how the treatments effectiveness is verified in clinical reality, this methodology was chosen to conduct the present study.

There is sufficient evidence available regarding the role of fluoride in preventing or arresting WSLs progression.^6,31^ Nevertheless, they may remain clinically visible as most of the detection signals comes from the lesion body, which cannot be completely remineralized.^7^ In this study, it was noted that the remineralization with sodium fluoride neutral gel in high concentration was able to mask the WSL in relation to the adjacent sound enamel. This can be justified by the enamel mineral gain, which is directly related to its translucency.^32^ Furthermore, according to Jones and Fried^7^(2006), who evaluated the WSL reflexivity after immersion in a fluoride solution, the esthetic improvement of the lesion is not only related to its mineral gain, but also to the directional nature of the repair, related to the disposition of the crystals.

Nonetheless, these results differ from those found in the *in vitro* study by Torres, et al.^25^(2011) Although the fluoride-enhanced remineralization protocol used was the same, the color assessment compared the WSL color with that of the adjacent sound surface. It is also important to emphasize that the lesions used in the present study were artificial, and as such were shallower compared to their natural counterparts. Thus, the results might differ in deeper lesions.

Moreover, in the present study, the treatment with resin infiltrant was also able to mask the WSL in relation to the adjacent sound enamel. This is because the colorless material basically consists of TEGDMA, has a refractive index close to that of hydroxyapatite (1.52), and replaces the water or air present in the lesion pores.^9^ As such, the whitish appearance of the lesion almost disappears, and it becomes roughly imperceptible in relation to the surrounding structure. This is called the “chameleon effect,” since the resin infiltrant does not act through color matching.^9,33^

Groups in which WSLs were treated with fluoride-enhanced remineralization and resin infiltration not followed by bleaching presented medians of total color change (ΔE_00_ of 1.84 and 2.07, respectively) above the perceptibility (0.8) and acceptability (1.8) thresholds.^19^ This means that for half the observers, this color difference, even though visible, may be acceptable, while the other half may find it unacceptable. In this context, if patients are still dissatisfied with their teeth color, even after remineralization or resin infiltration, subsequent tooth bleaching may be pertinent.

However, the effect of bleaching after resin infiltration needs to be better elucidated, since the infiltrant could be a blocking barrier.^30^ This is why in the present study bleaching was performed not only on the infiltrated area, but on the entire specimen surface. The ΔE_00_ values found after bleaching showed that the WSLs remained indistinguishable from the adjacent sound enamel. This can be explained by similarities with other studies that show the effectiveness of tooth bleaching during orthodontic treatment. Once the hydrogen peroxide has a low molecular weight and high diffusibility, successful bleaching can be achieved even in the presence of some blocking agent.^34^

It is important to highlight that when bleaching was performed, the medians of total color change (ΔE_00_ of 1.58 and 1.50, respectively) were situated between the color difference perceptibility (0.8) and acceptability (1.8) thresholds. Thus, considering a clinical situation, if the patient still notices some difference in color after remineralization or infiltration of the lesion, bleaching could be a valid alternative to reduce discomfort.

Furthermore, it is worth mentioning that not even a single tooth presents a homogeneous color, but rather a color gradation that varies according to the enamel and dentin thickness. This color variation within the same tooth could be verified from the control group specimens, as the derived ΔE_00_ median (1.63) exceeded the color difference perceptibility threshold.

In addition to the esthetic improvement provided by the treatments presented, it is important to evaluate their impact on enamel surface properties, such as surface roughness. In this study, fluoride-enhanced remineralization, followed or not by bleaching, was unable to make the surface roughness of WSLs similar to that of the adjacent enamel, at the same level found between adjacent areas of the control specimens.

Other studies have shown that the resin infiltrant is able to reduce the WSL roughness, but not to reach the values of a sound enamel.^35^ Conversely, in this study, the WSL surface roughness and its respective treatments were evaluated comparatively to their adjacent sound surface. Therefore, we observed that the infiltrated WSLs presented surface roughness much similar to that of the adjacent sound surface, which can be explained by the polishing of the infiltrant surface with rubber cups.^36^

Bleaching, however, apparently suppressed the effect of resin infiltration in minimizing the surface roughness difference between the WSL and the adjacent sound enamel, which is consistent with previous research indicating an increase in surface roughness after bleaching in resin-based materials.^37^ Perhaps this increase in roughness could not be processed *in vivo* due to salivary flow and fluoride availability.^37,38^

This study had similar limitations to other *in vitro* studies, so clinical extrapolations should be considered with care. Bovine teeth were used, and the carious lesions were artificial, which tend to be more superficial than the natural ones,^39^ besides not presenting a classical dark zone, richer in organic content. Furthermore, only one kind of fluoride-enhanced remineralization protocol, as well as only one kind of bleaching were considered, not to mention the fact that staining and re-bleaching were not considered. However, most studies in resin infiltration using artificial carious lesions do not validate whether they are subsurface,^25,27,28,30^ which this study carefully did. The present results are thus valuable in encouraging further investigation of bleaching as a complement to fluoride-enhanced remineralization or resin infiltration in masking WSLs.

## Conclusion

Both fluoride-enhanced remineralization and resin infiltration, followed by bleaching or not, were able to mask WSLs. However, subsequent bleaching may be an interesting option to reduce the color differences bellow the acceptability threshold, even though it can suppress the favorable effects of resin infiltration regarding enamel surface roughness.
